# [^18^F]Fluorodeoxyglucose positron emission tomography ([ ^18^F]FDG PET) Characterizes Neurodegeneration Levels Across the *α‐*Synucleinopathy Continuum

**DOI:** 10.1002/mds.70301

**Published:** 2026-04-05

**Authors:** Beatrice Orso, Ignacio Roura, Pietro Mattioli, Francesco Famà, Federico Massa, Luigi Lorenzini, Andrea Brugnolo, Nicola Girtler, Mattia Losa, Stefano Raffa, Luca Sofia, Monica Roascio, Gabriele Arnulfo, Silvia Morbelli, Matteo Pardini, Dario Arnaldi

**Affiliations:** ^1^ Department of Neuroscience, Rehabilitation, Ophthalmology, Genetics, Maternal and Child Health (DINOGMI) University of Genoa Genoa Italy; ^2^ Medical Psychology Unit, Department of Medicine, Institute of Neurosciences University of Barcelona Barcelona Spain; ^3^ Institut d'Investigacions Biomèdiques August Pi i Sunyer (IDIBAPS) Barcelona Spain; ^4^ Clinical Neurophysiology IRCCS Azienda Ospedaliera Metropolitana Genoa Italy; ^5^ Clinical Neurology Unit IRCCS Azienda Ospedaliera Metropolitana Genoa Italy; ^6^ Department of Radiology and Nuclear Medicine Amsterdam UMC Location VuMC, Vijre Universiteit Amsterdam, Amsterdam Neuroscience Amsterdam The Netherlands; ^7^ Clinical Psychology Unit IRCCS Azienda Ospedaliera Metropolitana Genoa Italy; ^8^ Department of Health Science (DISSAL) University of Genoa Genoa Italy; ^9^ Nuclear Medicine Unit IRCCS Azienda Ospedaliera Metropolitana Genoa Italy; ^10^ Department of Informatics, Bioengineering, Robotics and System Engineering (DIBRIS) University of Genoa Genoa Italy; ^11^ Nuclear Medicine Unit AOU Città Della Salute e Della Scienza di Torino Turin Italy; ^12^ Department of Medical Sciences University of Turin Turin Italy

**Keywords:** [18F]FDG PET, disease progression, neurodegeneration, neuroimaging, α‐synucleinopathy

## Abstract

**Background:**

[^18^F]Fluorodeoxyglucose positron emission tomography ([^18^F]FDG PET) represents an endorsed neurodegeneration biomarker in neuronal *α‐*synucleinopathies. Idiopathic/isolated rapid eye movement (REM) sleep behavior disorder (iRBD) represents a prodromal stage of such disorders.

**Objectives:**

To assess [^18^F]FDG PET as a neurodegeneration biomarker, using published brain metabolic disease‐related patterns, and a regional‐based approach, across the prodromal to overt *α‐*synucleinopathy continuum.

**Methods:**

We included 83 prodromal subjects with iRBD, comprising non‐converters (n = 56) and converters (n = 27) to an overt *α‐*synucleinopathy (either Parkinson's disease [PD] or dementia with Lewy bodies [DLB]) according to the last available follow‐up, and 85 subjects with PD (n = 40) and DLB (n = 45). For comparison, we enrolled a group of healthy subjects (n = 41). Participants underwent brain [^18^F]FDG PET at baseline. Analysis of covariance was used to test the ability of previously published [^18^F]FDG PET disease‐related patterns in characterizing neurodegeneration levels along the prodromal to overt *α‐*synucleinopathy continuum, and across the motor‐predominant (parkinsonism‐first) and the cognitive‐predominant (dementia‐first) clinical trajectories. We further assessed metabolic changes using a regional‐based approach.

**Results:**

All disease‐related patterns effectively discriminated clinical stages, from prodromal to overt *α*‐synucleinopathies, with comparable performance. [^18^F]FDG PET significantly distinguished all groups along the cognitive‐predominant pathway; whereas in the motor‐predominant pathway, converter patients were not significantly discriminated from non‐converters. Regionally, the inferior parietal, precuneus, and middle frontal areas exhibited the most prominent decrease in [^18^F]FDG uptake with progression, alongside relative parallel progressive increases in the cerebellum, pons, parahippocampal areas, putamen, and pallidum.

**Conclusions:**

[^18^F]FDG PET disease‐related patterns efficiently characterize neurodegeneration from prodromal to overt *α*‐synucleinopathy, best assessing the cognitive‐predominant (dementia‐first) pathway. © 2026 The Author(s). *Movement Disorders* published by Wiley Periodicals LLC on behalf of International Parkinson and Movement Disorder Society.

Neuronal *α*‐synucleinopathies, namely Parkinson's disease (PD) and dementia with Lewy bodies (DLB), are characterized by a prolonged prodromal period, in which non‐motor symptoms, such as idiopathic/isolated rapid eye movement (REM) sleep behavior disorder (iRBD) and hyposmia, may precede overt neurological decline by years.^1^, ^2^ Recently, new frameworks for biological definition and staging systems in *α*‐synucleinopathies have been proposed.^3^, ^4^ Reliable biomarkers able to characterize neurodegeneration across the continuum from prodromal to overt neuronal *α*‐synucleinopathy are needed to translate the new frameworks into clinical practice.

[^18^F]Fluorodeoxyglucose positron emission tomography ([^18^F]FDG PET), enabling the study of brain glucose metabolic abnormalities at the network level (PD‐related pattern, PD‐RP), has been endorsed as a neurodegeneration imaging biomarker in PD.^3^, ^5^, ^6^, ^7^, ^8^ However, the neuronal *α*‐synucleinopathy continuum includes at least two main clinical trajectories, that is, a motor‐predominant (parkinsonism‐first) and a cognitive‐predominant (dementia‐first) phenotype. Thus, other, more specific patterns have been identified, such as de novo PD with RBD‐RP (PDRBD‐RP)^9^, ^10^ and de novo DLB with RBD‐RP (DLBRBD‐RP).^9^ Another pattern specific for short‐term phenoconversion in iRBD, regardless of the outcome diagnosis (ie, PD or DLB), has also been identified (iRBDconv‐RP).^11^ While all these patterns are associated with phenoconversion in iRBD patients, their relative effectiveness to characterize neurodegeneration along the *α*‐synucleinopathy continuum remains to be explored. Indeed, it is unclear whether all patterns equally track neurodegeneration in both motor‐predominant and cognitive‐predominant trajectories.

It should be noted that all these disease‐related patterns partially overlap in the topography of relative brain glucose hypermetabolism and hypometabolism. Of note, the disease‐related patterns are obtained through covariance analyses, namely Scaled Subprofile Model of Principal Component Analysis (SSM‐PCA), which enables the derivation of relative contrasts across brain regions within a single component. This approach has the advantage of allowing both hypometabolic and hypermetabolic regions to co‐occur within a mathematically defined framework, thereby providing a general overview of the main metabolic changes.^12^ However, these global spatial patterns may mask divergent metabolic changes in specific regions over time as the disease progresses. Therefore, a region‐based approach may provide extra information about disease progression that may be overlooked by network approaches.

In this study, we investigated the ability of previously described brain metabolic disease‐related patterns – iRBDconv‐RP, PDRBD‐RP, DLBRBD‐RP, and PD‐RP – to characterize neurodegeneration across the neuronal *α*‐synucleinopathy continuum, as well as in the specific clinical phenotypes (ie, cognitive‐ and motor‐predominant). Additionally, we aimed to assess metabolic changes across disease stages using two complementary, region‐based approaches: (i) analyzing hypermetabolic and hypometabolic global masks separately and (ii) examining changes among commonly affected regions of interest (ROIs). Our overall goal was to assess the potential of [^18^F]FDG PET as a biomarker of neurodegeneration levels along the prodromal to overt neuronal *α*‐synucleinopathy continuum.

## Patients and Methods

### Patients

Patients were enrolled in IRCCS Policlinico San Martino, Genova, Italy between January 2013 and December 2024. Inclusion criteria were a diagnosis of iRBD, DLB, and PD,^2^, ^13^, ^14^ confirmed by evidence of dopaminergic deficit on [^123^I]FP‐CIT SPECT and by at least 2 years of clinical follow‐up. Presence of RBD in DLB and in PD patients was assessed by video‐polysomnography (vPSG).^15^ Furthermore, only drug‐naïve subjects, at the time of [^18^F]FDG PET, were included.

Exclusion criteria for all participants were the presence of other brain diseases (such as focal lesions), assessed through brain magnetic resonance imaging (MRI) or computed tomography (CT),^16^, ^17^ as well as the presence of white matter hyperintensities if the Wahlund scale was >1 for each brain region.^18^ Information on clinical assessment is described in detail in the Supplementary Materials.

A total of 168 patients (aged 72 ± 7.01 years, 67.9% males) along the prodromal to overt neuronal *α*‐synucleinopathy continuum were enrolled, including 83 iRBD patients (71 ± 7 years, 74.69% males), representing the prodromal stage, and 85 PD and DLB patients (40 PD, 72 ± 7.4 years, 57.5% males, and 45 DLB, 76 ± 5.78 years, 64.4% males), representing the overt stage. All iRBD patients were followed over time (27 ± 24 months) to investigate phenoconversion, namely the emergence of overt PD^19^ or DLB^13^ clinical syndromes. According to the follow‐up outcome, the iRBD group was retrospectively stratified as non‐converters (ncRBD, n = 56, 70 ± 6.95 years, 75.0% males), if patients did not develop overt parkinsonism or dementia, or converters (cRBD, n = 27, 73 ± 6.87 years, 74.1% males). The latter group was furtherly stratified as PD converters (cRBD‐PD, n = 11, 72 ± 8.86 years, 54.5% males) or DLB converters (cRBD‐DLB, n = 16, 74 ± 5 years, 87.5% males) patients, according to the follow‐up diagnosis. To identify PD converters, we applied the 1‐year rule, that is none among the PD converters developed dementia within 1 year since phenoconversion diagnosis.

For comparison, we enrolled a group of healthy controls (HC, n = 41, 70 ± 8.68 years, 36.6% males). Their cognitive status was assessed with a Mini‐Mental State Examination (MMSE) score >27 and Clinical Dementia Rating scale (CDR) = 0.

Therefore, we defined two disease progression frameworks, with increasing granularity: one with three levels (HC, prodromal, and overt stages) and another with four levels (HC, ncRBD, cRBD, and overt stage). We assume that these levels of stratification, applied based on clinical features, likely reflect the stages of neurodegeneration along the continuum from prodromal to overt *α*‐synucleinopathy.^20^ Additionally, we divided the entire cohort into two pathways: a motor‐predominant pathway (HC, ncRBD, cRBD‐PD, and overt PD) and a cognitive‐predominant pathway (HC, ncRBD, cRBD‐DLB, and overt DLB), to investigate the different clinical trajectories along the continuum. These frameworks are summarized in Figure S1.

All patients underwent [^18^F]FDG PET within 12 months from diagnosis, in accordance with international guidelines,^21^ to investigate brain glucose metabolism. Acquisition methods were as previously described^9^, ^22^, ^23^ and are briefly reported, together with video‐polysomnography recording methods, in the Supplementary Materials.

### Disease‐Related Patterns: Derivation and Application

Three disease‐related patterns previously described by our group were used in this study: the iRBDconvRP,^11^ the PDRBD‐RP,^9^ and the DLBRBD‐RP.^9^

Moreover, using the SSM‐PCA algorithm,^12^, ^24^ we identified a fourth pattern (the PD‐RP), from the [^18^F]FDG PET of 32 PD patients (69 ± 9 years, 53% males) not included in the previous studies, and 41 HC (70 ± 8.68 years, 36.6% males). Derivation methods are reported elsewhere^9^, ^11^ and are briefly summarized in the Supplementary Materials. Notably, our PD‐RP largely overlaps with the published PD‐RP, as is known from replication literature studies.^6^

Each disease‐related pattern was separately applied on the [^18^F]FDG PET scans of the participants, both along the two models of granularity (three‐ and four‐levels), and clinical trajectories (motor‐ and cognitive‐predominant). A single‐subject score was obtained, reflecting the degree of expression of each disease pattern (RBDconv‐RP, PDRBD‐RP, DLBRBD‐RP, and PD‐RP) within each group. Subject scores of each pattern were then z‐transformed in respect to the HC group.

### Relative Hypometabolic and Hypermetabolic Masks

Given the high degree of overlap across the patterns, we built a global hypermetabolic mask, comprising the following ROIs: the anterior cingulate cortex (ACC), cerebellum, pallidum, parahippocampal cortex, pons, post‐central gyrus, putamen, and thalamus. Conversely, the global hypometabolic mask included the inferior parietal gyrus, inferior temporal gyrus, inferior lingual gyrus, middle frontal gyrus, posterior cingulate cortex (PCC), and the precuneus. Detailed mask definition and [^18^F]FDG PET standardized uptake value ratios (SUVRs) extraction are described in the Supplementary Materials.

## Standard Protocol Approval

All patients provided written consent prior to study participation. In cases where the patient did not show the capacity of consent, the legal caregiver was asked to sign the consent form. The local ethics committee approved the study protocol (CER Liguria: 105/2023‐DB id 13,027), and all participants signed an informed consent form in compliance with the Helsinki Declaration of 1975.

### Study Overview and Statistical Analysis

Between‐group differences in clinical characteristics were compared using univariate analysis of variance (ANOVA) tests (normally distributed) or the Kruskal–Wallis test (non‐normally distributed), using Status as the independent variable. Normal distribution of variables was checked using the Shapiro–Wilk test. Categorical variables were compared using *χ* ^2^ or Fisher's exact tests.

As a first step, an analysis of covariance (ANCOVA) was performed on z‐transformed subject scores to test differences in pattern expression across each level of stratification and clinical trajectory, using age and sex as covariates, with the aim of performing a head‐to‐head comparison between the disease‐related patterns.

As a second step, we used a regional‐based approach for discriminating across levels of stratification and clinical trajectories. Thus, an ANCOVA was performed on the SUVRs of both the hypermetabolic and the hypometabolic global masks, separately, using age and sex as covariates, for each level of stratification and clinical trajectory. Furthermore, to explore regional differences, two separate multivariate ANCOVAs were performed on the SUVRs of each hypermetabolic and hypometabolic ROI. Following significant multivariate ANOVA results, univariate ANCOVAs were conducted for each regional SUVR measure, including Status as a fixed factor and Age and Sex as covariates. Post‐hoc comparisons between Status groups were performed using pairwise contrasts of estimated marginal means (least‐squares means), with Tukey's adjustment applied to control for multiple comparisons.

Lastly, to study whether there was an association between pairs of hypermetabolic and hypometabolic ROIs, and whether these differed significantly across clinical groups (ie, group‐dependent correlation), we performed interaction analyses, using linear regression models. For each combination of ROIs, linear models were fitted, using each hypermetabolic ROI as the dependent variable, and each hypometabolic ROI, clinical group (Status), and their interaction term as predictors. The resulting *P*‐values were corrected for multiple comparisons using false discovery rate (FDR).

Statistical threshold was set at 0.05, and *P*‐values were reported using FDR corrected for multiple comparisons. All analyses were performed using MatLab (version 2020a; MathWorks, Natick, MA, USA) and R (R Core Team, 2020)‐RStudio (Rstudio Team, 2020).

## Results

### Group Characteristics

Demographic and clinical differences across each level of stratification and clinical trajectories are described and reported in the Supplementary Materials and Tables S1–S4.

### Disease‐Related Patterns Performance

**FIG. 1:**
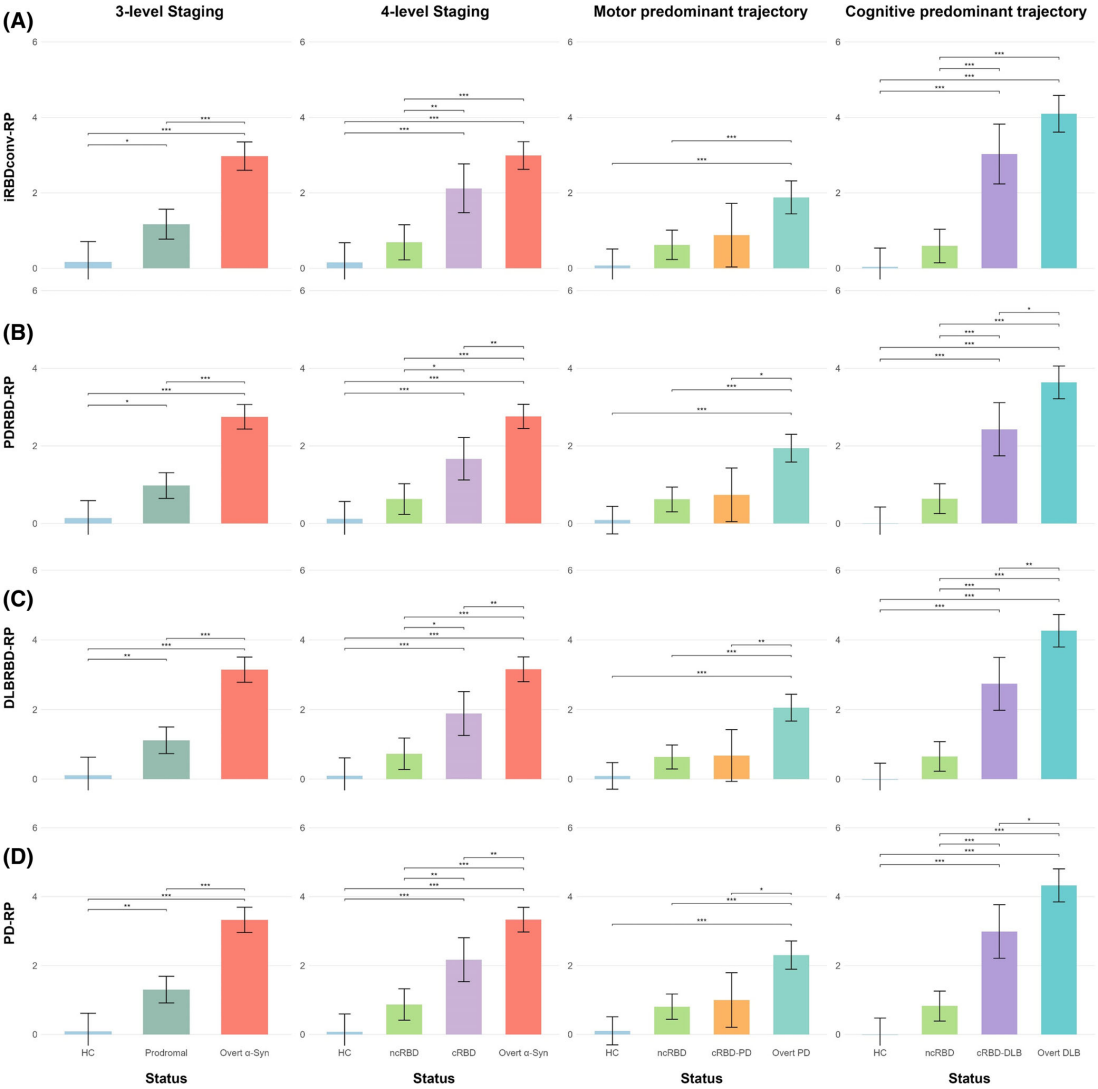
Disease‐related patterns expression across disease stages and clinical trajectories. Discrimination performance of the (A) idiopathic/isolated rapid eye movement (REM) sleep behavior disorder (iRBD) conversion‐related pattern (iRBD‐RP); (B) Parkinson's disease (PD) with RBD‐related pattern (PDRBD‐RP); (C) dementia with Lewy bodies (DLB) with RBD‐related pattern (DLBRBD‐RP); and (D) PD‐related pattern (PD‐RPHC). HC, healthy controls; *α*‐Syn, *α*‐synucleinopathy; ncRBD, non‐converters RBD; cRBD, converters RBD. *P < 0.5, **P < 0.01, ***P < 0.001. [Color figure can be viewed at wileyonlinelibrary.com]

ANCOVA analyses revealed that disease‐related patterns showed significantly different expression along the stratification levels and clinical trajectories (Fig. 1). Notably, at the four‐level staging, all patterns significantly distinguished between cRBD and ncRBD status. Conversely, none of the patterns significantly differentiated cRBD‐PD from ncRBD patients within the motor‐predominant pathway, while all patterns were able to discriminate cRBD‐DLB from ncRBD within the cognitive‐predominant pathway. Additionally, all patterns significantly discriminated cRBD patients from overt stages patients, along all stratification levels and clinical trajectories, except for the iRBDconvRP. Details of post‐hoc results and statistics for each pattern are reported in Table 1 and in the Supplementary Materials. Bootstrapped 95% confidence intervals of *η* ^2^ largely overlapped across disease‐related patterns, indicating comparable discrimination strength across staging frameworks (Table S8).

**TABLE 1:** Post‐hoc analyses of metabolic disease‐related patterns' performance.

Disease‐related metabolic patterns' performance				
Disease‐related Pattern	Frameworks (df)	*T* values	Contrasts	*P*‐value
iRBDconv‐RP	Three‐level (203)	−2.905	Prodromal > HC	<0.05
		−8.244	*α*‐Syn > HC	<0.001
		−6.613	*α*‐Syn > Prodromal	<0.0001
	Four‐level (202)	−4.585	cRBD > HC	0.001
		−8.573	*α*‐Syn > HC	<0.0001
		−3.6	cRBD > ncRBD	<0.01
		−7.699	*α*‐Syn > ncRBD	<0.0001
	Motor‐predominant pathway (151)	−5.379	Overt PD > HC	<0.001
		−4.291	Overt PD > ncRBD	<0.001
	Cognitive‐predominant pathway (151)	−6.202	cRBD‐DLB > HC	<0.001
		−5.382	cRBD‐DLB > ncRBD	<0.001
		−5.282	Overt DLB > HC	<0.001
		−10.515	Overt DLB > ncRBD	<0.001
PDRBD‐RP	Three‐level (203)	−9.157	*α*‐Syn > HC	<0.0001
		−7.735	*α*‐Syn > Prodromal	<0.0001
		−2.917	Prodromal > HC	<0.05
	Four‐level (202)	−4.26	cRBD > HC	0.0002
		−9.42	*α*‐Syn > HC	<0.0001
		−3.09	cRBD > ncRBD	0.012
		−8.43	*α*‐Syn > ncRBD	<0.0001
		−3.47	*α*‐Syn > cRBD	0.0035
	Motor‐predominant pathway (141)	−7.204	Overt PD > HC	<0.001
		−5.505	Overt PD > ncRBD	<0.001
		−3.083	Overt PD > cRBD‐PD	<0.05
	Cognitive‐predominant pathway (151)	−11.559	Overt DLB > HC	<0.001
		−10.402	Overt DLB > ncRBD	<0.001
		−3.045	Overt DLB > cRBD	<0.05
		−5.839	cRBD‐DLB > HC	<0.001
		−4.568	cRBD‐DLB > ncRBD	<0.001
DLBRBD‐RP	Three‐level (203)	−3.029	Prodromal > HC	<0.01
		−9.253	*α*‐Syn > HC	<0.001
		−7.713	*α*‐Syn > Prodromal	<0.001
	Four‐level (202)	−4.278	cRBD > HC	0.001
		−9.505	*α*‐Syn > HC	0.0001
		−2.986	cRBD > ncRBD	0.016
		−8.356	*α*‐Syn > ncRBD	<0.0001
		−3.52	*α*‐Syn > cRBD	0.002
	Motor‐predominant pathway (141)	−7.080	Overt PD > HC	<0.001
		−5.489	Overt PD > ncRBD	<0.001
		−3.274	Overt PD > cRBD‐PD	<0.01
	Cognitive‐predominant pathway (151)	−12.256	Overt DLB > HC	<0.001
		−11.301	Overt DLB > ncRBD	<0.001
		−3.464	Overt DLB > cRBD	<0.01
		−5.967	cRBD‐DLB > HC	<0.001
		−4.804	cRBD‐DLB > ncRBD	<0.001
PD‐RP	Three‐level (203)	−3.597	Prodromal > HC	<0.01
		−9.787	*α*‐Syn > HC	<0.001
		−7.623	*α*‐Syn > Prodromal	<0.001
	Four‐level (202)	−3.173	*α*‐Syn > cRBD	<0.01
		−10.077	*α*‐Syn > HC	<0.0001
		−8.470	*α*‐Syn > ncRBD	<0.0001
		−3.328	cRBD > ncRBD	<0.01
		−4.957	cRBD > HC	<0.0001
	Motor‐predominant pathway (141)	−7.446	Overt PD > HC	<0.001
		−5.429	Overt PD > ncRBD	<0.001
		−2.909	Overt PD > cRBD‐PD	<0.05
	Cognitive‐predominant pathway (151)	−12.153	Overt DLB > HC	<0.001
		−10.727	Overt DLB > ncRBD	<0.001
		−2.979	Overt DLB > cRBD	<0.05
		−6.351	cRBD‐DLB > HC	<0.001
		−4.875	cRBD‐DLB > ncRBD	<0.001

Abbreviations: cRBD, converters RBD; df, degrees of freedom; DLB, dementia with Lewy bodies; DLBRBD‐RP, dementia with Lewy bodies (DLB) with RBD‐related pattern (DLBRBD‐RP); HC, healthy controls; *α*‐Syn, *α*‐synucleinopathy; iRBDconRP, idiopathic/isolated rapid eye movement (REM) sleep behavior disorder (iRBD) conversion‐related pattern; ncRBD, non‐converters RBD; PD, Parkinson's disease; PDRBD‐RP, Parkinson's disease (PD) with RBD‐related pattern.

#### iRBDconv‐RP

Analyses of covariance showed significant intergroup differences in iRBDconv‐RP expression found at both three‐ and four‐level staging (*F*(2,203) = 52.146, *P* < 0.0001, *η* ^2^ = 0.325; *F*(3,202) = 46.286, *η* ^2^ = 0.373, *P* < 0.0001, respectively), with large effect size.

Regarding clinical trajectories, significant intergroup differences in pattern expression were found across the motor‐ and cognitive‐predominant pathways (*F*(3,141) = 14.059, *P* < 0.0001, *η* ^2^ = 0.215; *F*(3,151) = 64.452, *P* < 0.0001, *η* ^2^ = 0.560, respectively). Results are shown in Figure 1A.

#### denovoPDRBD‐RP

Analyses of covariance yielded significant intergroup differences in denovoPDRBD‐RP expression at both three‐ and four‐level staging, with large effect sizes (*F*(2,203) = 62.851, *P* < 0.0001, *η* ^2^ = 0.453; *F*(3,202) = 47.38, *P* < 0.0001, *η* ^2^ = 0.401, respectively).

Regarding clinical trajectories, analyses of covariance yielded significant intergroup differences in pattern expression across the motor‐ and cognitive‐predominant pathways, showing a greater effect size in the latter (*F*(3,141) = 21.882, *P* < 0.0001, *η* ^2^ = 0.299; *F*(3,151) = 61.805, *P* < 0.0001, *η* ^2^ = 0.549, respectively). Results are shown in Figure 1B.

#### denovoDLBRBD‐RP

Analyses of covariance showed that significant intergroup differences in DLBRBD‐RP expression were found at both three‐ and four‐level staging (*F*(2,203) = 62.231, *P* < 0.0001, *η* ^2^ = 0.373; *F*(3,202) = 46.626, *η* ^2^ = 0.404, *P* < 0.0001, respectively), with large effect size.

Regarding clinical trajectories, significant intergroup differences in pattern expression were found across the motor‐ and cognitive‐predominant pathways (*F*(3,141) = 21.185, *P* < 0.0001, *η* ^2^ = 0.294; *F*(3,151) = 69.466, *P* < 0.0001, *η* ^2^ = 0.577, respectively) with large effect size. Results are shown in Figure 1C.

#### PD‐RP

Analyses of covariance yielded significant intergroup differences in PD‐RP expression were found at both three‐ and four‐level staging (*F*(2,203) = 62.951, *η* ^2^ = 0.378, *P* < 0.0001; *F*(3,202) = 48.095, *η* ^2^ = 0.413, *P* < 0.0001, respectively), with large effect size.

Regarding clinical trajectories, significant intergroup differences in pattern expression were found across the motor‐ and cognitive‐predominant pathways (*F*(3,141) = 23.021, *P* < 0.0001, *η* ^2^ = 0.311; *F*(3,151) = 67.085, *P* < 0.0001, *η* ^2^ = 0.568, respectively), with large effect size. Results are shown in Figure 1D.

### Relative Hypometabolic and Hypermetabolic Global Mask Performance

**FIG. 2:**
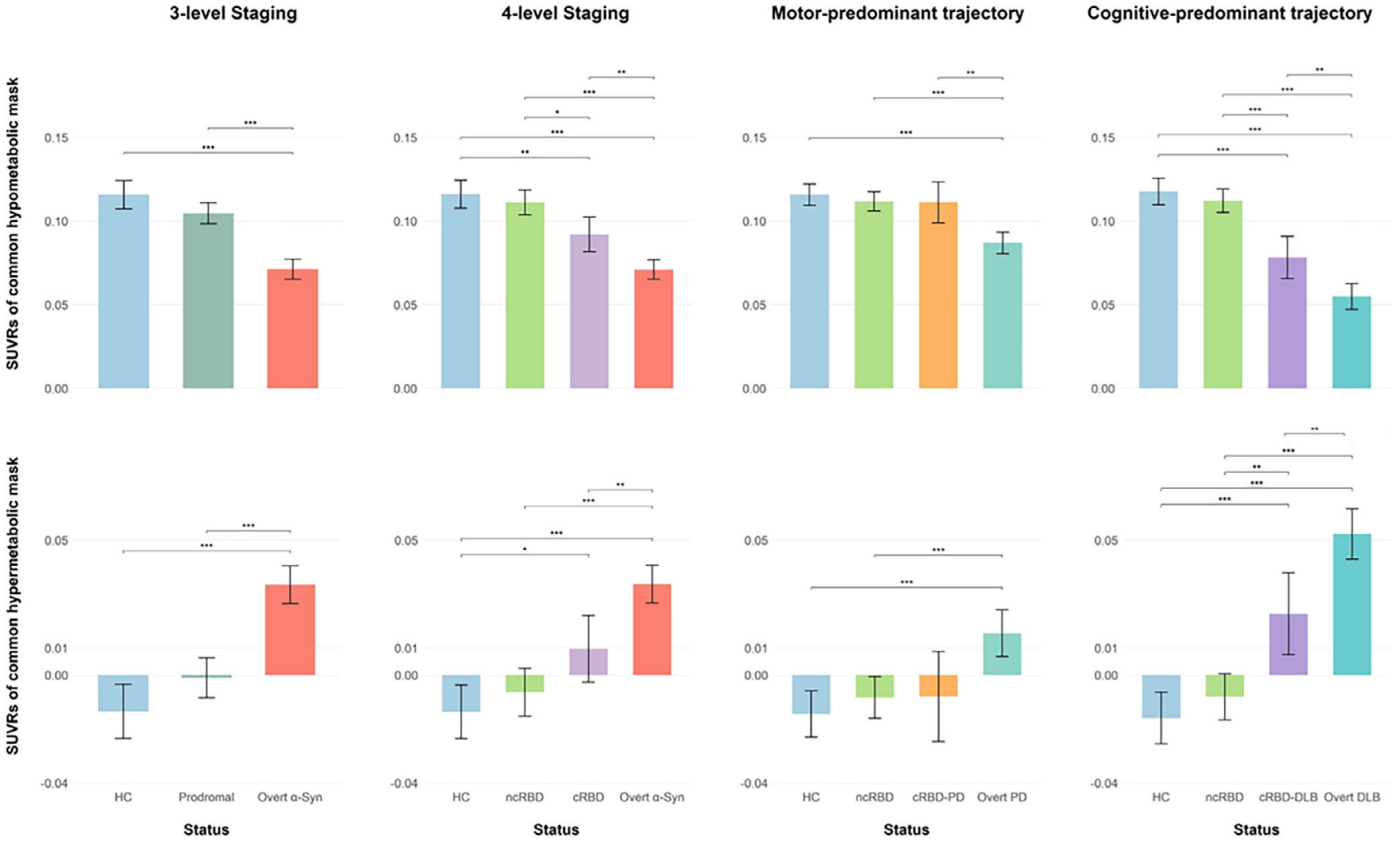
Metabolism of common hypometabolic and hypermetabolic regions of interest (ROIs) across disease stages and clinical trajectories. SUVR, standardized uptake value ratio; HC, healthy controls; *α*‐Syn, *α*‐synucleinopathy; ncRBD, non‐converters rapid eye movement (REM) sleep behavior disorder; cRBD, converters RBD; PD, Parkinson's disease; DLB, dementia with Lewy bodies. *P < 0.5, **P < 0.01, ***P < 0.001. [Color figure can be viewed at wileyonlinelibrary.com]

Analyses of covariance yielded significant intergroup differences in both the SUVRs of common relative hypometabolic and hypermetabolic masks at the three‐level staging (*F*(2,205) = 51.48, *η* ^2^ = 0.33, *P* < 0.0001; *F*(2,205) = 43.49, *η* ^2^ = 0. 3, *P* < 0.0001, respectively); four‐level staging (*F*(3,204) = 38.93, *η* ^2^ = 0.36, *P* < 0.0001; *F*(3,204) = 31.78, *η* ^2^ = 0.32, *P* < 0.0001, respectively); the motor‐predominant pathway (*F*(3,143) = 17.19, *η* ^2^ = 0.26, *P* < 0.0001; *F*(3,143) = 9.91, *η* ^2^ = 0.17, *P* < 0.0001, respectively); and the cognitive‐predominant pathway (*F*(3,1453) = 53.74, *η* ^2^ = 0.51, *P* < 0.0001; *F*(3,153) = 46.71, *η* ^2^ = 0.48, *P* < 0.0001, respectively). Results are shown in Figure 2.

The hypometabolic global mask significantly differentiated almost every study group, including ncRBD from cRBD and cRBD from overt stages in the four‐level staging and the cognitive‐predominant pathway. Still, it did not distinguish ncRBD from cRBD‐PD within the motor‐predominant pathway. Conversely, the hypermetabolic global mask differentiated most study groups, including ncRBD from cRBD‐DLB within the cognitive‐predominant pathway. However, it did not discriminate between ncRBD and cRBD in the four‐level stratification, nor between cRBD and cRBD‐PD in the motor‐predominant pathway.

Results of detailed post‐hoc analyses are reported in the Supplementary Materials.

### Relative Hypometabolic and Hypermetabolic ROIs Performance

Regional analysis showed significant intergroup differences in hypometabolic and hypermetabolic ROIs across stratification levels and clinical trajectories. Details of post‐hoc analysis and statistics are reported in Tables S6 and S7.

**FIG. 3:**
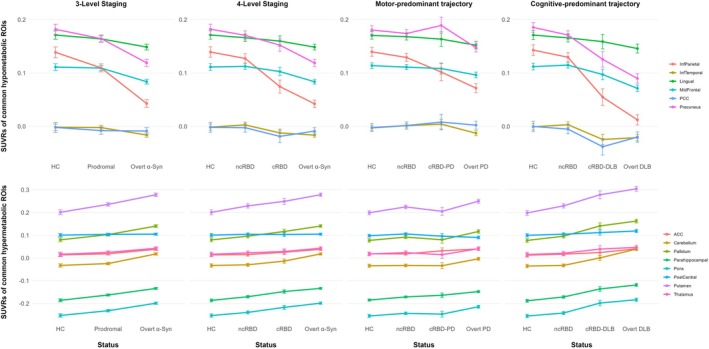
Metabolism of common hypometabolic and hypermetabolic regions of interest (ROIs) across disease stages and clinical trajectories. SUVR, standardized uptake value ratio; HC, healthy controls; *α*‐Syn, *α*‐synucleinopathy; ncRBD, non‐converters rapid eye movement (REM) sleep behavior disorder; cRBD, converters RBD; PD, Parkinson's disease; DLB, dementia with Lewy bodies; Inf, inferior; Mid, middle; PCC, posterior cingulate cortex; ACC, anterior cingulate cortex. *P < 0.5. [Color figure can be viewed at wileyonlinelibrary.com]

The relative hypometabolic ROIs, in the three‐ and four‐level staging, showed a significant change in [^18^]FDG uptake in the middle frontal, inferior parietal, and precuneus areas, with moderate to large effect sizes; in the cognitive‐predominant pathway, together with the aforementioned regions, a significant change in [^18^]FDG uptake was also observed in the inferior temporal area. In the motor‐predominant pathway, the inferior parietal and the precuneus areas showed significant change across stages (Fig. 3).

As for the relative hypermetabolic ROIs, progressive significant changes in [^18^]FDG uptake were seen in the cerebellum, pallidum, parahippocampal, putamen, and pons areas, across all disease stages and clinical trajectories, all with moderate to large effect sizes (Figure 3).

**FIG. 4:**
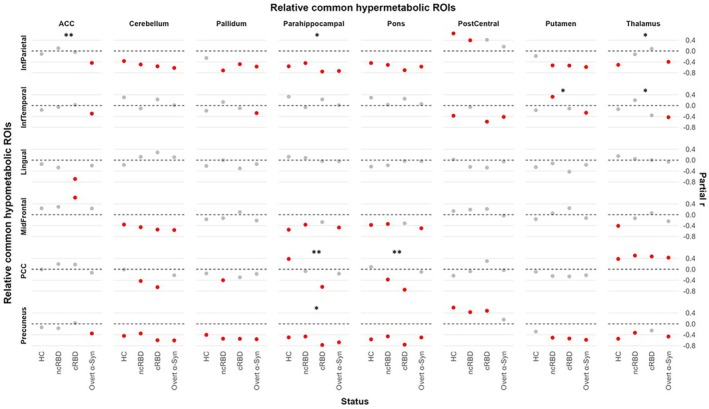
Interregional partial correlations between common relative hypermetabolic and hypometabolic standardized uptake value ratios (SUVRs) across disease groups of the four‐level stratification. Red points represent significant correlations that survived false discovery rate (FDR)‐correction for multiple comparisons (age‐ and sex‐adjusted). Asterisks indicate region of interest (ROI) pairs showing a significant Hypometabolic × Status interaction, based on linear models testing whether the association between regions differed across disease stages. ACC, anterior cingulate cortex; *uncorrected *P* < 0.05 for interaction term; **FDR‐corrected *P* < 0.05 for interaction term; Inf, inferior; Mid, middle; HC, healthy controls; PCC, posterior cingulate cortex; ncRBD, non‐converters RBD; *α*‐Syn, *α*‐synucleinopathy; cRBD, converters RBD. [Color figure can be viewed at wileyonlinelibrary.com]

We furtherly investigated potential changes in the associations of hypermetabolic and hypometabolic ROIs across stratification levels and clinical trajectories. For the four‐level stratification, linear models revealed significant interaction effects for the pairs PCC‐Parahippocampal (*F* = 7.127, *P* = 0.006), PCC‐Pons (*F* = 6.296, *P* = 0.010) and InfParietal‐ACC (*F* = 4.929, *P* = 0.040), suggesting that the associations between these pairs of regions significantly changed with disease progression (Figure 4). Results of potential changes in the associations of common hypermetabolic and hypometabolic ROIs in the three‐level stratification, motor‐ and cognitive‐predominant pathways are reported in the Supplementary Materials.

## Discussion

In the present study, we assessed the performance of brain [^18^F]FDG PET in characterizing neurodegeneration levels in subjects across the prodromal to overt *α*‐synucleinopathy continuum by applying both network analysis and a region‐based approach. The first finding is that all previously published disease‐related patterns significantly track the clinical stages along the synucleinopathy continuum, with similar performances.

To note, the statement of similar performance across disease‐related patterns should be interpreted in a descriptive sense, suggesting no clear evidence of differential discriminatory performance.

The PD‐RP has been proposed as an endorsed biomarker for the biological classification of PD.^3^ Cumulative evidence has shown that PD‐RP is expressed already at prodromal stages (ie, iRBD), and that its expression is associated with clinical features and the risk of phenoconversion.^10^, ^25^, ^26^, ^27^, ^28^ However, original PD‐RPs were obtained from PD patients without specified RBD presence.^8^, ^29^, ^30^ Subsequent studies have derived more specific disease‐related patterns from PD and DLB patients with evidence of RBD at baseline,^9^, ^10^ as well as a phenoconversion‐related pattern (namely the iRBDconv‐RP),^11^ with the aim of better understanding metabolic features of the neuronal *α*‐synucleinopathy spectrum. The results of our study showed that all these patterns performed similarly in tracking the progressive clinical stages along the prodromal to overt neuronal *α*‐synucleinopathy continuum. Thus, the currently endorsed and most widely used PD‐RP may serve as a practical disease‐related pattern to be considered for patient stratification in upcoming disease‐modifying clinical trials.

Remarkably, while all patterns significantly distinguished between patients at all stages along the cognitive‐predominant pathway, none of them could differentiate between prodromal patients who eventually developed PD (cRBD‐PD) and non‐converters patients in the motor‐predominant pathway. Whereas it could be hypothesized that this is due to the limited sample size of the cRBD‐PD group, the fact that the cRBD‐DLB group, with a similar sample size, was significantly differentiated from the other groups, could point towards a distinct metabolic signature underlying both phenotypes. In this line, it is well‐established that brain cortical metabolic alterations are more severe in DLB compared with PD, which instead exhibit more prominent nigrostriatal degeneration.^9^, ^31^, ^32^, ^33^ Indeed, a recent multicenter study show dopamine transporter‐single‐photon emission computed tomography (DaT‐SPECT) can effectively stage patients in the motor‐predominant pathway.^20^ Altogether, [^18^F]FDG PET may serve as a suitable biomarker to study neurodegeneration levels across cognitive‐predominant phenotypes, while progression along the motor‐predominant trajectory may be better assessed using dopaminergic imaging techniques such as [^123^I]FP‐CIT SPECT (DaT‐SPECT).

The region‐based analyses substantially confirmed these two main results. The methodology used for disease‐related patterns derivation (SSM‐PCA) allows one to obtain relative contrasts across brain regions within a single component, where both hypometabolic and hypermetabolic regions co‐occur in a mathematically defined framework.^12^ By using global masks of hypermetabolism and hypometabolism, it becomes possible to quantify rates of metabolic changes. Indeed, both global masks significantly characterized patients along the prodromal to overt *α*‐synucleinopathy continuum, especially those in the cognitive‐predominant pathway. Conversely, cRBD‐PD patients were not significantly different from non‐converters patients in the motor‐predominant pathway.

Interestingly, using a region‐based approach, we showed that among all the common hypometabolic regions, the inferior parietal, precuneus, and middle frontal areas exhibited the most prominent decrease in [^18^F]FDG uptake with disease progression. Notably, these are common regions characterizing patients lying in both the motor‐predominant and the cognitive‐predominant pathway. Thus, it is not surprising that the well‐known parieto‐occipital relative hypometabolism, which is specific to DLB patients, is not included.

In parallel, we observed a progressive increase in [^18^F]FDG uptake in the cerebellum, pons, parahippocampal areas, putamen, and pallidum. Our findings partially align with studies using whole‐brain voxel‐based approaches. Indeed, decreased metabolism in the medial parietal and occipital structures has consistently been reported in individuals with iRBD,^34^, ^35^, ^36^ together with hypermetabolism in the brainstem^34^ and preserved metabolism in the posterior cingulate cortex.^35^, ^36^ Recent studies indicate that while PCC hypometabolism may not be observed in iRBD,^35^, ^36^ a faster decline in its metabolic activity occurs as progression advances to overt dementia‐first phenotypes, namely DLB.^35^ In our study, we found that PCC metabolism did not significantly change across disease stages. However, progressively stronger negative associations emerged between PCC metabolism and that of typically hypermetabolic areas, such as the parahippocampal gyrus and the pons, only in the prodromal stages. These findings may indicate a compensatory role of these subcortical structures in response to early cortical hypometabolism,^36^, ^37^ while later in the disease course these associations may weaken, reflecting the possible failure of compensatory mechanisms due to progressive pathological burden.^36^, ^37^

Our study comes with both limitations and strengths. First, whereas we aimed to study the progression of metabolic changes across the *α*‐synucleinopathy continuum, not all patients included in this study had biologically confirmed neuronal synucleinopathies based on in vivo biomarkers, such as seed amplification assay in cerebrospinal fluid analysis or skin biopsy. Additionally, the pseudoprogression design of our study allows the comparison of patients in different disease stages but does not permit the assessment of longitudinal progression at the individual level. The lack of significant differences between the cRBD‐PD and ncRBD groups should be interpreted cautiously. The relatively small size of the cRBD‐PD subgroup (n = 11), together with the conservative multiple‐comparison correction applied in the post‐hoc analyses, may have limited the statistical power to detect group differences. Consequently, this finding should be regarded as preliminary and warrants validation in larger cohorts. Nevertheless, the significant difference observed between the cRBD‐DLB and ncRBD groups, despite the limited sample size, suggests that brain [^18^F]FDG PET may better characterize patients belonging to the cognitive‐predominant (dementia‐first) alpha‐synucleinopathy pathway, rather than the motor‐predominant (parkinsonism‐first) pathway. Brain [^18^F]FDG PET is among the most promising outcome measures for disease‐modifying trials in iRBD cohorts, and the PD‐RP has been selected as the primary endpoint in the PRISMS study (https://clinicaltrials.gov/study/NCT06996652). Our results suggest that there is the risk that the PD‐RP would better capture the disease progression of patients developing a cognitive‐predominant phenotype rather than a motor‐predominant one.

Lastly, it has to be acknowledged that, in the present study, we are not exploring the performance of the MSA‐RP^31^ in our cohorts, and some of our patients might phenoconvert to multiple system atrophy (MSA) in the coming years.

Our study has the strength of having assessed the ability of previously published metabolic disease‐related patterns in discriminating among different disease stages and clinical pathways along the *α*‐synucleinopathy continuum. Additionally, our study included a well‐characterized cohort of patients in the overt stage of *α*‐synucleinopathy, in which RBD was present before clinical diagnosis. Taken together, our results highlight the need for future longitudinal studies with larger sample sizes, possibly incorporating confirmation of *α*‐synuclein pathology.

In conclusion, all the previously published disease‐related patterns performed similarly in discriminating disease stages from prodromal to overt *α*‐synucleinopathy. Brain [^18^F]FDG PET could be considered a particularly sensitive biomarker in tracking the progression along the cognitive‐predominant pathway.

## Author Roles

(1) Research Project: A. Conception, B. Organization, C. Execution; (2) Statistical Analysis: A. Design, B. Execution, C. Review and Critique; (3) Manuscript Preparation: A. Writing of the First Draft, B. Review and Critique.

B.O.: 1A, 1B, 1C, 2A, 2B, 3A.

I.R.: 1A, 1B, 1C, 2A, 2B, 3A.

P.M.: 1A, 1B, 1C, 2A, 2B, 3A.

F.F.: 1C, 2C, 3B.

F.M.: 1C, 2C, 3B.

L.L.: 1C, 2C, 3B.

A.B.: 1C, 2C, 3B.

N.G: 2C, 3B.

M.L.: 1C, 2C, 3B.

S.R.: 1C, 2C, 3B.

L.S.: 1C, 2C, 3B.

M.R.: 1C, 2C, 3B.

G.A.: 1C, 2C, 3B.

S.M.: 1C, 2C, 3B.

M.P.: 1C, 2C, 3B.

D.A.: 1A, 1B, 1C, 2A, 2B, 2C, 3A, 3B.

## Financial Disclosures of All Authors (for the Past 12 Months)

This work was supported by: NEXTGENERATIONEU (NGEU), funded by the Ministry of University and Research (MUR), National Recovery and Resilience Plan (NRRP), project MNESYS (PE0000006) A Multiscale Integrated Approach to the Study of the Nervous System in Health and Disease (DN. 1553 11.10.2022) for the activities of F.M. #NEXTGENERATIONEU (NGEU), funded by the European Union, NRRP M6C2–Investment 2.1 Enhancement and Strengthening of Biomedical Research in the NHS (PNRR‐MCNT2‐2023‐12377527‐CUP C13C23001070006) for the activities of M.P. Bando Ricerca Finalizzata RF‐2021‐12374240, funded by the Italian Ministry of Health, for the activities of D.A. S.M. was supported by a grant from the Italian Ministry of University and Research (BANDO PRIN 2022 Prot. 2022WK7NHC). The views and opinions expressed are those of the authors alone and do not necessarily reflect those of the European Union or the European Commission. Neither the European Union nor the European Commission can be held responsible for the views and opinions expressed herein.

## Supporting information


**Table S1.** Demographic and clinical characteristics of the three‐level staging. Continuous variables are shown as mean ± standard deviation; median [range]. Categorical variables are shown as number (percentage).
**Table S2.** Demographic and clinical characteristics of the four‐level staging. Continuous variables are shown as mean ± standard deviation; median [range]. Categorical variables are shown as number (percentage).
**Table S3.** Demographic and clinical characteristics of the motor‐predominant pathway. Continuous variables are shown as mean ± standard deviation; median [range]. Categorical variables are shown as number (percentage).
**Table S4.** Demographic and clinical characteristics of the cognitive‐predominant pathway. Continuous variables are shown as mean ± standard deviation; median [range]. Categorical variables are shown as number (percentage).
**Table S6.** Post‐hoc results of regional analysis of the [^18^F]fluorodeoxyglucose ([^18^F]FDG) uptake within the common hypermetabolic regions of interest, across disease stages and clinical trajectories. Effect sizes of significant test (*P* < 0.05) are reported in bold type.
**Table S7.** Post‐hoc results of regional analysis of the [^18^F]fluorodeoxyglucose ([^18^F]FDG) uptake within the common hypometabolic regions of interests, across disease stages and clinical trajectories. Effect sizes of significant test (*P* < 0.05) are reported in bold type.
**Table S8.** Bootstrapped 95% confidence intervals of *η* ^2^ for the effect of disease stage across disease‐related [^18^F]fluorodeoxyglucose positron emission tomography ([^18^F]FDG PET) patterns. Confidence intervals were estimated using bootstrap resampling and show substantial overlap across patterns within each staging framework and clinical trajectory, supporting comparable discriminatory performance.
**Figure S1.** Framework of granularity along the continuum from prodromal to overt *α*‐synucleinopathy.
**Figure S2.** Interregional partial correlations between common relative hypermetabolic and hypometabolic standardized uptake value ratios (SUVRs) across disease groups of the (A) three‐level stratification; (B) motor‐predominant pathway; and (C) cognitive‐predominant pathway. Red dots represent significant correlations that survived false discovery rate (FDR)‐correction for multiple comparisons (age‐ and sex‐adjusted).

## Data Availability

The data that support the findings of this study are available from the corresponding author upon reasonable request.
