# Supplementary material for: [18F]Fluorodeoxyglucose positron emission tomography ([ 18F]FDG PET) Characterizes Neurodegeneration Levels Across the α‐Synucleinopathy Continuum
Source: Mov Disord. 2026 Apr 5;41(7):1786–97. doi: 10.1002/mds.70301 (PMC13387997; doi:10.1002/mds.70301)
Supplement: Supplementary file 1 — Table S1. Demographic and clinical characteristics of the three‐level staging. Continuous variables are shown as mean ± standard deviation; median [range]. Categorical variables are shown as number (percentage). Table S2. Demographic and clinical characteristics of the four‐level staging. Continuous variables are shown as mean ± standard deviation; median [range]. Categorical variables are shown as number (percentage). Table S3. Demographic and clinical characteristics of the motor‐predominant pathway. Continuous variables are shown as mean ± standard deviation; median [range]. Categorical variables are shown as number (percentage). Table S4. Demographic and clinical characteristics of the cognitive‐predominant pathway. Continuous variables are shown as mean ± standard deviation; median [range]. Categorical variables are shown as number (percentage). Table S6. Post‐hoc results of regional analysis of the [18F]fluorodeoxyglucose ([18F]FDG) uptake within the common hypermetabolic regions of interest, across disease stages and clinical trajectories. Effect sizes of significant test (P < 0.05) are reported in bold type. Table S7. Post‐hoc results of regional analysis of the [18F]fluorodeoxyglucose ([18F]FDG) uptake within the common hypometabolic regions of interests, across disease stages and clinical trajectories. Effect sizes of significant test (P < 0.05) are reported in bold type. Table S8. Bootstrapped 95% confidence intervals of η 2 for the effect of disease stage across disease‐related [18F]fluorodeoxyglucose positron emission tomography ([18F]FDG PET) patterns. Confidence intervals were estimated using bootstrap resampling and show substantial overlap across patterns within each staging framework and clinical trajectory, supporting comparable discriminatory performance. Figure S1. Framework of granularity along the continuum from prodromal to overt α‐synucleinopathy. Figure S2. Interregional partial correlations between common relative hypermetabolic and hypome [file MDS-41-1786-s001.docx]

**[^18^F]FDG-PET characterizes neurodegeneration levels across the α-synucleinopathy continuum**

Beatrice Orso, PhD^1*^; Ignacio Roura, PsyD^2,3*^; Pietro Mattioli, MD^1,4^; Francesco Famà, BSc^1,4^; Federico Massa, MD, PhD^1,5^; Luigi Lorenzini, PhD^1,6^; Andrea Brugnolo, PhD^1,7^; Nicola Girtler, PsyD^1,7^; Mattia Losa, MD^1^; Stefano Raffa, MD^8,9^; Luca Sofia, MD^8^; Monica Roascio, PhD^10^; Gabriele Arnulfo, PhD^10^; Silvia Morbelli, MD, PhD^11,12^; Matteo Pardini, MD, PhD^1,5^; Dario Arnaldi, MD, PhD^1,4^

1 Department of Neuroscience, Rehabilitation, Ophthalmology, Genetics, Maternal and Child Health (DINOGMI), University of Genoa, Genoa, Italy.

2 Medical Psychology Unit, Department of Medicine, Institute of Neurosciences, University of Barcelona, Barcelona, Catalonia, Spain.

3 Institut d’Investigacions Biomèdiques August Pi i Sunyer (IDIBAPS), Barcelona, Catalonia, Spain.

4 Clinical Neurophysiology, IRCCS Azienda Ospedaliera Metropolitana, Genoa, Italy.

5 Clinical Neurology Unit, IRCCS Azienda Ospedaliera Metropolitana, Genoa, Italy.

6 Department of Radiology and Nuclear Medicine, Amsterdam UMC Location VuMC, Vijre Universiteit Amsterdam, Amsterdam Neuroscience, Amsterdam, 1081 HV, The Netherlands.

7 Clinical Psychology Unit, IRCCS Azienda Ospedaliera Metropolitana, Genoa, Italy.

8 Department of Health Science (DISSAL), University of Genoa, Genoa Italy.

9 Nuclear Medicine Unit, IRCCS Azienda Ospedaliera Metropolitana, Genoa, Italy.

10 Department of Informatics, Bioengineering, Robotics and System Engineering (DIBRIS),

University of Genoa, Genoa, Italy.

11 Nuclear Medicine Unit, AOU Città Della Salute e Della Scienza di Torino, Turin, Italy.

12 Department of Medical Sciences, University of Turin, Turin, Italy.

*Authors equally contributed

**Corresponding Author:** Beatrice Orso, PhD. Department of Neuroscience, Rehabilitation, Ophthalmology, Genetics, Maternal and Child Health (DINOGMI), University of Genoa, Largo Daneo 3, 16132, Genoa, Italy. E-mail: [beatrice27orso@gmail.com](mailto:beatrice27orso@gmail.com)

# Methods

## Patients

At baseline, all patients underwent the Movement Disorder Society Unified Parkinson Disease rating scale, third part (MDS-UPDRS-III) to quantify motor impairment, the Mini Mental State Examination (MMSE), as a measure of global cognitive functioning, as well as a comprehensive neuropsychological assessment, including at least two tests for each of the main cognitive domains (verbal memory, executive functions, attention and working memory, visuospatial abilities and language)^1^, to evaluate the presence of mild cognitive impairment (MCI).^2^ Clinical information on activities and instrumental activities of daily living assessment, motor and cognitive assessment were evaluated at baseline and prospectively every six months.

## [^18^F]FDG-PET and Video Polysomnography Recording

A SIEMENS Biograph 16 PET/CT hybrid system with a total axial field of view of 15 cm and no interplane gap space was used. To ensure blood glucose levels remained below 7.8 mmol/L, patients were required to fast for at least six hours prior to the examination. Once blood glucose levels were confirmed, patients rested for 10 minutes in a quiet, darkened room with their eyes closed and ears unplugged. Subsequently, 185–250 MBq of [^18^F]FDG was administered via a venous cannula. Patients remained in the same environment for 30 minutes post-injection before being transferred to the PET room. PET scanning commenced 15 minutes later and lasted for ten minutes. A low-dose computed tomography scan was also performed to enable attenuation correction. The acquired data were reconstructed using an ordered subset expectation maximization (OSEM) algorithm with 16 subsets and 6 iterations, resulting in a reconstructed voxel size of 1.33 × 1.33 × 2.00 mm. All [^18^F]FDG-PET images were acquired in static mode and subsequently underwent affine and nonlinear spatial normalization into Montreal Neurological Institute (MNI) space using SPM12 (Wellcome Department of Cognitive Neurology, London, UK). All the default settings of SPM were applied, with a specific [^18^F]FDG-PET brain template serving as the reference.31 The spatially normalized images were then smoothed using a 10-mm isotropic Gaussian filter to accommodate individual anatomical differences and enhance the signal-to-noise ratio.

Sleep scoring was performed following current criteria.^3^ PSG derivations were placed according to recommended rules^3–5^ to evaluate sleep features, respiratory, cardiac, and limb events. Chin electromyography (EMG) was used to visually quantify REM sleep without atonia (RWA) and the ‘any’ REM percentage was used.^6,7^ If used, patients were asked to withdraw melatonin, hypnotic medications and antidepressant drugs for two weeks before the recording.

# Disease Related Patterns: derivation and application

Disease-related patterns were derived by applying an automated algorithm, based on the SSM-PCA method^8^ implemented in Matlab (version 2020a; MathWorks, Natick, MA), to the [^18^F]FDG-PET data of each disease group. The components that could best discriminate between controls and patients are linearly combined to form a disease-related pattern.^9^ For validation, a leave-one-out cross validation (LOOCV) is performed, along with a bootstrap resampling (1000 repetitions) to extract the most stable regions in the patterns found**.**

To determine the sensitivity and specificity of the PD-RP, a receiver operating curve (ROC) was plotted based on z-transformed subject scores from both patterns.

## Identification of Overlapping Brain Regions

The ROIs were created using WFU_PickAtlas (<https://www.nitrc.org/projects/wfu_pickatlas/>) implemented in MatLab (version 2020a; MathWorks, Natick, MA).

Furthermore, the identified ROIs were merged into two global masks, representing the hypermetabolic global mask and the hypometabolic global mask, respectively.

Using the MarsBar 0.44 region of interest toolbox (<https://marsbar-toolbox.github.io/>) for SPM12, we extracted the [^18^F]FDG PET Standardized Uptake Value Ratios (SUVRs) of each single hypometabolic and hypermetabolic ROIs, as well as of the two global masks. We then normalized the SUVRs over the whole brain as follows: (ROI average uptake - whole brain average uptake)/whole brain average uptake.

# Results

## Group **characteristics**

In the 3-level staging, Overt α-synucleinopathy patients were older (F=5.594; p=0.004) and had a lower MMSE score (F=23.165; p<0.001) compared to both HC and Prodromal patients (Supplementary Table 1).

In the 4-level staging, Overt α-synucleinopathy patients were older (F=4.893; p<0.001) and had a lower MMSE score (F=16.288; p<0.001) compared to both HC and ncRBD patients and had a more severe motor impairment (F=91.359; p<0.001) compared to both ncRBD and cRBD patients. Moreover, cRBD patients performed worst at the MMSE compared to HC (Supplementary Table 2).

When exploring clinical trajectories, we found that, in the motor-predominant pathway, Overt PD patients had a worst MMSE (F=4.309; p=0.006) compared to HC and a more severe motor impairment (F=122.479; p<0.001) compared to both ncRBD and cRBD (Supplementary Table 3). In the cognitive-predominant pathway, Overt DLB patients were older (F=7.915;p<0.001) than both HC and ncRBD; had a lower MMSE score (F=35.897; p<0.001) compared to HC, ncRBD and cRBD and had a more severe motor impairment (F=53.389; p<0.001) compared to both ncRBD and cRBD. cRBD patients also exhibit a worst MMSE score (F=35.897; p<0.001) compared to HC (**Supplementary Table 4**).

**Supplementary Table 1:** Demographic and clinical characteristics of the 3-level staging. Continuous variables are shown as mean±standard deviation; median [range]. Categorical variables are shown as number (percentage).

|  | **Healthy Controls**  **(n=41)** | **Prodromal (n=83)** | **Overt α-synucleinopathy**  **(n=85)** | **Statistic** | **p-value** |
| --- | --- | --- | --- | --- | --- |
| Age | 70±8.64 [71] | 71±7 [71] | 74±6.86 [74] | 5.594 F​ | 0.004^b,c​^ |
| Sex | 15:26 [M:F] | 65:21 [M:F] | 52:33 [M:F] | 18.041 χ^2^​ | <0.001^a,b​^ |
| Education | 11±3.8 [11] | 11±4 [8] | 10±4.19 [10] | 0.171 F​ | 0.842​ |
| MMSE | 29±0.7 [29] | 28±2.3 [29] | 26±3.9 [27] | 23.165 F​ | <0.001^b,c​^ |
| MDS-UPDRS III | / | 2±2.9 [1] | 19±10 [16] | 1378U​ | <0.001​ |
| Survival Time* | / | 27±24 [22] | / | / | / |

*Legend: at Tukey post-hoc analysis, a = HC vs. Prodromal; b = HC vs. α-synucleinopathy; c = Prodromal vs. α-synucleinopathy; MDS-UPDRS-III = Movement Disorders Society-sponsored revision of the Unified Parkinson’s Disease Rating Scale, motor section; MMSE = Mini Mental State Examination*

|  | **Healthy Controls**  **(n=41)** | **Non converters RBD (n=56)** | **Converters RBD**  (n=27) | **Overt α-synucleinopathy**  **(n=85)** | **Statistic​** | **p-value​** |
| --- | --- | --- | --- | --- | --- | --- |
| Age | 70±8.64 [71] | 70±6.95 [70] | 73±6.84 [74] | 74±6.86 [74] | 4.893 F​ | <0.001^c,d^ |
| Sex | 15:26 [M:F] | 42:14 [M:F] | 23:7 [M:F] | 52:33 [M:F] | 18.064 χ^2^​​ | <0.001^a,b​^ |
| Education | 11±3.8 [11] | 11±4.19 [11] | 10±4.22 [8] | 10±4.19 [10] | 0.212 F​ | 0.888​ |
| MMSE | 29±0.7 [29] | 28±1.6 [29] | 27±3.1 [29] | 26±3.9 [27] | 16.288 F​ | <0.001^b,c,d​^ |
| MDS-UPDRS III | / | 2±1.9 [1] | 3±4.1 [2] | 19±10 [16] | 91.359 F​ | <0.001^d,e​^ |
| Survival Time* | / | 30±25.28 [24] | 23±21.38 [18] | / | 820 U​ | <0.001​ |

**Supplementary Table 2:** Demographic and clinical characteristics of the 4-level staging. Continuous variables are shown as mean±standard deviation; median [range]. Categorical variables are shown as number (percentage).

*Legend: at Tukey post-hoc analysis, a = HC vs. ncRBD; b = HC vs. cRBD; c = HC vs. α-synucleinopathy; d = ncRBD vs. α-synucleinopathy; e = cRBD vs. α-synucleinopathy; MDS-UPDRS-III = Movement Disorders Society-sponsored revision of the Unified Parkinson’s Disease Rating Scale, motor section; MMSE = Mini Mental State Examination*

**Supplementary Table 3:** Demographic and clinical characteristics of the Motor-predominant pathway. Continuous variables are shown as mean±standard deviation; median [range]. Categorical variables are shown as number (percentage).

|  | **Healthy Controls**  **(n=41)** | **Non converters RBD (n=56)** | **RBD-PD converters**  **(n=11)** | **Overt Parkinson’s Disease**  **(n=40)** | **Statistic​** | **p-value​** |
| --- | --- | --- | --- | --- | --- | --- |
| Age | 70±8.64 [71] | 70±6.95 [70] | 72±8.3 [73] | 72±7.43 [73] | 0.692 F | 0.558 |
| Sex | 15:26 [M:F] | 42:14 [M:F] | 9:5 [M:F] | 23:17 [M:F] | 14.634 χ^2^​​ | 0.002^a^ |
| Education | 11±3.8 [11] | 11±4.19 [11] | 11±4 [8] | 11±3.95 [11] | 0.273 F | 0.844 |
| MMSE | 29±0.7 [29] | 28±1.6 [29] | 28±2.6 [29] | 28±2.1 [29] | 4.309 F | 0.006^b^ |
| MDS-UPDRS III | / | 2±1.9 [1] | 4±4.1 [3] | 20±8.8 [17] | 122.479 F | <0.001^c,d^ |
| Survival Time* | / | 30±25.28 [24] | 26±19.45 [26] | / | 2346 U | <0.001 |

*Legend: at Tukey post-hoc analysis, a = HC vs. ncRBD; b = HC vs. Overt PD; c = ncRBD vs. Overt PD; d = cRBD vs. Overt PD; MDS-UPDRS-III = Movement Disorders Society-sponsored revision of the Unified Parkinson’s Disease Rating Scale, motor section; MMSE = Mini Mental State Examination*

**Supplementary Table 4:** Demographic and clinical characteristics of the Cognitive-predominant pathway. Continuous variables are shown as mean±standard deviation; median [range]. Categorical variables are shown as number (percentage).

|  | **Healthy Controls**  **(n=41)** | **Non converters RBD (n=56)** | **RBD-DLB converters**  **(n=16)** | **Overt Dementia with Lewy Bodies**  **(n=45)** | **Statistic​** | **p-value​** |
| --- | --- | --- | --- | --- | --- | --- |
| Age | 70±8.64 [71] | 70±6.95 [70] | 74±5 [75] | 76±5.78 [77] | 7.915 F | <0.001^c,d^ |
| Sex | 15:26 [M:F] | 42:14 [M:F] | 14:2 [M:F] | 29:16 [M:F] | 19.952 χ^2^​​ | <0.001^a,b^ |
| Education | 11±3.8 [11] | 11±4.19 [11] | 10±4 [8] | 9±4.26 [8] | 1.012 F | 0.389 |
| MMSE | 29±0.7 [29] | 28±1.6 [29] | 27±4 [28] | 24±4 [24] | 35.897 F | <0.001^b,c,d,e^ |
| MDS-UPDRS III | / | 2±1.9 [1] | 2±4 [1] | 18±12.5 [16] | 53.389 F | <0.001^d,e^ |
| Survival Time* | / | 30±25.28 [24] | 20±23.19 [16] | / | 2485 U | <0.001 |

*Legend: at Tukey post-hoc analysis, a = HC vs. ncRBD; b = HC vs. cRBD-DLB; c = HC vs. Overt-DLB; d = ncRBD vs. Overt-DLB; e = cRBD vs. Overt-DLB; MDS-UPDRS-III = Movement Disorders Society-sponsored revision of the Unified Parkinson’s Disease Rating Scale, motor section; MMSE = Mini Mental State Examination*

## Disease-related Patterns Performance

*iRBDconv-RP*

Post-hoc analyses in the 3-level staging revealed greater pattern expression in the Prodromal (p<0.05) and Overt α-Syn (p<0.001) groups compared to HC. Moreover, Overt α-Syn patients showed greater pattern expression compared to the Prodromal group (p<0.001).

In the 4-level, compared to HC, greater pattern expression was found in both cRBD (p=0.001) and Overt α-Syn (p<0.0001). Additionally, both cRBD and Overt α-Syn patients exhibited greater pattern expression relative to ncRBD (p<0.01; p<0.0001, respectively).

Within the motor-predominant pathway, post-hoc analyses showed greater pattern expression in the Overt PD group, relative to both HC (p<0.001) and ncRBD (p<0.001) groups. In the cognitive-predominant pathway, we found an increased pattern expression in cRBD-DLB relative to HC (p<0.001) and ncRBD (p<0.001) patients, as well as in Overt DLB patients compared to HC (p<0.001) and ncRBD (p<0.001) patients.

*denovoPDRBD-RP*

In detail, post-hoc analyses in the 3-level staging showed greater pattern expression in Overt α-Syn patients compared to both HC (p<0.0001) and Prodromal (p<0.0001) groups. The latter showed greater pattern expression relative to HC (p<0.05).

In the 4-level staging, HC showed lower pattern expression compared to cRBD (p=0.0002) and Overt α-Syn (p<0.0001) patients. Additionally, ncRBD patients exhibited a lower pattern expression compared to cRBD (p=0.012) and Overt α-Syn patients (p<0.0001). Moreover, Overt α-Syn patients showed greater pattern expression relative to cRBD (p=0.0035).

In the motor-predominant pathway, post-hoc analyses showed greater pattern expression in the Overt PD group relative to HC (p<0.001), ncRBD (p<0.001) and cRBD-PD (p<0.05) patients. Within the cognitive-predominant pathway, we found that Overt DLB patients exhibited greater pattern expression compared to HC (p<0.001), ncRBD (p<0.001) and cRBD-DLB (p<0.05). Moreover, cRBD-DLB showed an increased pattern expression relative to both HC (p<0.001) and ncRBD (p<0.001).

*denovoDLBRBD-RP*

At 3-level staging, post-hoc analyses revealed greater pattern expression relative to HC in the Prodromal (p<0.01) and Overt α-Syn (p<0.001) groups. Overt α-Syn patients showed greater pattern expression relative to the Prodromal group (p<0.001). In the 4-level, greater pattern expression was found in both cRBD (p=0.001), and Overt α-Syn (p=0.0001), compared to HC. Additionally, ncRBD patients showed lower pattern expression compared to cRBD patients (p=0.016) and Overt α-Syn patients (p<0.0001). The latter group showed increased pattern expression compared to cRBD (p=0.002).

In the motor-predominant pathway, post-hoc analyses showed greater pattern expression in the Overt PD group relative to HC (p<0.001), ncRBD (p<0.001) and cRBD-PD (p<0.01). Within the cognitive-predominant pathway, we found an increased pattern expression in Overt DLB patients compared to HC (p<0.001), ncRBD (p<0.001) and cRBD-DLB (p<0.01). Moreover, cRBD-DLB patients exhibited greater pattern expression relative to HC (p<0.001) and ncRBD (p<0.001).

*PD-RP*

Post-hoc analyses in the 3-level revealed greater pattern expression relative to HC in the Prodromal (p<0.01) and Overt α-Syn (p<0.001) groups. Overt α-Syn patients showed greater pattern expression relative to the Prodromal group (p<0.001). In the 4-level staging, greater pattern expression was found in the Overt α-Syn stage compared to HC (p<0.0001), ncRBD (p<0.0001) and cRBD (<0.01). Additionally, cRBD patients exhibited greater pattern expression relative to both HC (p<0.0001) and ncRBD (p<0.01).

As for the motor-predominant pathway, post-hoc analyses showed a significantly greater pattern expression in the Overt PD group relative to HC (p<0.001), ncRBD (p<0.001) and cRBD (p<0.05). Within the cognitive-predominant pathway, pattern expression was higher in the Overt DLB stage compared to HC (p<0.001), ncRBD (p<0.001) and cRBD-DLB (p<0.05). Moreover, cRBD-DLB exhibited higher pattern expression compared to HC (p<0.001) and ncRBD (p<0.001).

## Relative Hypometabolic and Hypermetabolic global mask Performance

In detail, post-hoc analysis in the 3-level staging revealed that Overt α-Syn exhibit a lower SUVRs uptake in the hypometabolic VOI compared to both HC (p<0.001) and Prodromal (p<0.001), as well as a higher SUVRs uptake in the hypermetabolic VOI compared to the same groups (HC: p<0.001; Prodromal: p<0.001, respectively).

In the 4-level staging, Overt α-Syn exhibit a lower SUVRs uptake in the hypometabolic VOI compared to HC (p<0.001), ncRBD (p<0.001) and cRBD (p<0.01); while cRBD had a lower SUVRs uptake compared to HC (p<0.01) and ncRBD (p<0.05). Additionally, α-Syn exhibit a higher SUVRs uptake in the hypermetabolic VOI compared to HC (p<0.001), ncRBD (p<0.001) and cRBD (p<0.01); while cRBD had a lower SUVRs uptake compared to HC (p<0.05).

When exploring clinical trajectories, in the motor-predominant pathway, Overt PD patients exhibit a lower SUVRs uptake in the hypometabolic VOI compared to HC (p<0.001), ncRBD (p<0.001) and cRBD-PD (p<0.01); as well as a higher SUVRs uptake in the hypermetabolic VOI compared to HC (p<0.001) and ncRBD (p<0.001).

Within the cognitive-predominant pathway, Overt DLB patients exhibit a lower SUVRs uptake in the hypometabolic VOI compared to HC (p<0.001), ncRBD (p<0.001) and cRBD-DLB (p<0.01); ); while cRBD-DLB had a lower SUVRs uptake compared to HC (p<0.001) and ncRBD (p<0.001). Additionally, Overt DLB patients showed a higher SUVRs uptake in the hypermetabolic VOI compared to HC (p<0.001), ncRBD (p<0.001) and cRBD-DLB (p<0.01); while cRBD-DLB had a higher SUVRs uptake compared to HC (p<0.001) and ncRBD (p<0.01).

## Relative Hypometabolic and Hypermetabolic ROIs Performance

Regional analysis showed significant intergroup differences in hypometabolic and hypermetabolic ROIs across disease stages and clinical trajectories. Details of post-hoc analysis and statistics are reported in **Supplementary Table 6** and **7**.

For the 3-level trajectory, linear models revealed significant interaction effects for the pairs PCC-Pons (F=7.949, p=0.023) and InfParietal-ACC (F=6.842, p=0.032), reflecting significant changes in their associations with disease progression. In the motor-predominant trajectory, no significant changes were observed (FDR-corrected) in the interregional associations across stages. In the cognitive-predominant trajectory, significant interaction effects were found for PCC-Parahippocampal (F=6.792, p=0.009), PCC-Pons (F=6.488, p=0.008), InfParietal-ACC (F=5.827, p=0.017), InfParietal-Parahippocampal (F=5.233, p=0.022), Precuneus-Parahippocampal (F=4.323, p=0.049) and InfParietal-Thalamus(F=4.296, p=0.049). (**Supplementary Figure 3A-B-C**)

**Supplementary Table 6:** Post-hoc results of regional analysis of the [^18^F]FDG uptake within the common hypermetabolic ROIs, across disease stages and clinical trajectories. Effect sizes of significant test (p<0.05) are reported in **bold**.

|  | **Relative Hypermetabolic Regions of Interest** | | | |
| --- | --- | --- | --- | --- |
|  | Region | F values | Contrasts | ŋ^2^ |
| **3-level Stratification** | ACC | 1.485 | ns | 0.012 |
|  | Cerebellum | 28.742 | α-Syn > HC = Prodromal | **0.212** |
|  | Pallidum | 27.665 | α-Syn > HC = Prodromal | **0.199** |
|  | Parahippocampal | 25.715 | α-Syn > Prodromal > HC | **0.201** |
|  | Postcentral | 0.3224 | ns | 0.003 |
|  | Putamen | 26.330 | α-Syn > Prodromal > HC | **0.194** |
|  | Pons | 28.598 | α-Syn > HC = Prodromal | **0.204** |
|  | Thalamus | 2.8710 | ns | 0.027 |
| **4-level Stratification** | ACC | 0.989 | ns | 0.012 |
|  | Cerebellum | 20.434 | α-Syn > HC, ncRBD, cRBD | **0.225** |
|  | Pallidum | 20.186 | α-Syn > HC, ncRBD; cRBD > HC | **0.216** |
|  | Parahippocampal | 19.364 | α-Syn > HC, ncRBD; cRBD > HC | **0.222** |
|  | Postcentral | 0.221 | ns | **0.227** |
|  | Putamen | 18.553 | α-Syn > HC, ncRBD; cRBD > HC | 0.003 |
|  | Pons | 21.576 | α-Syn > HC, ncRBD; cRBD > HC | **0.205** |
|  | Thalamus | 1.932 | ns | 0.028 |
| **Motor-predominant pathway** | ACC | 1.533 | ns | 0.026 |
|  | Cerebellum | 5.548 | Overt PD > HC, ncRBD | **0.1** |
|  | Pallidum | 6.266 | HC < Overt PD | **0.104** |
|  | Parahippocampal | 10.532 | Overt PD > HC, ncRBD | **0.178** |
|  | Postcentral | 0.878 | ns | **0.139** |
|  | Putamen | 8.058 | Overt PD > HC | 0.018 |
|  | Pons | 8.706 | Overt PD > HC, ncRBD | **0.128** |
|  | Thalamus | 1.492 | ns | 0.031 |
| **Cognitive-predominant pathway** | ACC | 0.388 | ns | 0.007 |
|  | Cerebellum | 27.782 | Overt DLB > HC, ncRBD, cRBD-DLB | **0.355** |
|  | Pallidum | 27.808 | cRBD-DLB > HC, ncRBD; Overt DLB > HC, ncRBD | **0.348** |
|  | Parahippocampal | 21.652 | cRBD-DLB > HC, ncRBD; Overt DLB > HC, ncRBD | **0.294** |
|  | Postcentral | 1.969 | ns | **0.34** |
|  | Putamen | 24.586 | cRBD-DLB > HC; Overt DLB > HC, ncRBD | 0.037 |
|  | Pons | 26.445 | cRBD-DLB > HC, ncRBD; Overt DLB > HC, ncRBD | **0.323** |
|  | Thalamus | 1.457 | ns | 0.028 |

*Legend: α-Syn = α-synucleinopathy; cRBD = converters RBD; DLB = Dementia with Lewy Bodies; HC = Healthy Controls; ncRBD = non-converters RBD; PD = Parkinson’s Disease; RBD = REM Sleep Behaviour Disorder; ACC = Anterior Cingulate Cortex; Inf = Inferior; Mid =Middle; PCC = Posterior cingulate Cortex.*

**Supplementary Table 7:** Post-hoc results of regional analysis of the [^18^F]FDG uptake within the common hypometabolic ROIs, across disease stages and clinical trajectories. Effect sizes of significant test (p<0.05) are reported in **bold**.

|  | **Relative Hypometabolic Regions of Interest** | | | |
| --- | --- | --- | --- | --- |
|  | Region | F values | Contrasts | ŋ^2^ |
| **3-level Stratification** | InfTemporal | 2.584 | ns | 0.024 |
|  | Lingual | 3.547 | ns | 0.034 |
|  | MidFrontal | 13.979 | α-Syn < HC, Prodromal | **0.118** |
|  | InfParietal | 43.401 | α-Syn < HC, Prodromal | **0.294** |
|  | PCC | 0.188 | ns | 0.002 |
|  | Precuneus | 20.306 | α-Syn < HC, Prodromal | **0.166** |
| **4-level Stratification** | InfTemporal | 2.421 | ns | 0.034 |
|  | Lingual | 2.454 | ns | 0.035 |
|  | MidFrontal | 9.955 | α-Syn < HC, ncRBD | **0.126** |
|  | InfParietal | 35.370 | cRBD < HC, ncRBD; α-Syn < HC, ncRBD | **0.34** |
|  | PCC | 0.677 | ns | 0.009 |
|  | Precuneus | 14.198 | α-Syn < HC, ncRBD | **0.174** |
| **Motor-predominant pathway** | InfTemporal | 1.789 | ns | 0.036 |
|  | Lingual | 1.417 | ns | 0.029 |
|  | MidFrontal | 2.678 | ns | 0.051 |
|  | InfParietal | 15.364 | Overt PD < HC, ncRBD | **0.236** |
|  | PCC | 0.134 | ns | 0.003 |
|  | Precuneus | 4.118 | Overt PD < HC | **0.079** |
| **Cognitive-predominant pathway** | InfTemporal | 3.005 | Overt DLB < ncRBD | **0.054** |
|  | Lingual | 1.903 | ns | 0.036 |
|  | MidFrontal | 13.482 | Overt DLB < HC, ncRBD | **0.21** |
|  | InfParietal | 41.825 | Overt DLB < HC, ncRBD, cRBD; cRBD-DLB < HC, ncRBD | **0.451** |
|  | PCC | 1.868 | ns | 0.035 |
|  | Precuneus | 21.558 | cRBD-DLB < HC; Overt DLB < HC, ncRBD | **0.296** |

*Legend: α-Syn = α-synucleinopathy; cRBD = converters RBD; DLB = Dementia with Lewy Bodies; HC = Healthy Controls; ncRBD = non-converters RBD; PD = Parkinson’s Disease; RBD = REM Sleep Behaviour Disorder. ACC = Anterior Cingulate Cortex; Inf = Inferior; Mid =Middle; PCC = Posterior cingulate Cortex.*

**Supplementary Table 8:** Bootstrapped 95% confidence intervals (CI) of η² for the effect of disease stage across disease-related [^18^F]FDG-PET patterns. Confidence intervals were estimated using bootstrap resampling and show substantial overlap across patterns within each staging framework and clinical trajectory, supporting comparable discriminatory performance.

| **Disease-related pattern** | **3-level staging η² (95% CI)** | **4-level staging η² (95% CI)** | **Motor-predominant pathway η² (95% CI)** | **Cognitive-predominant pathway η² (95% CI)** |
| --- | --- | --- | --- | --- |
| **DLBRBD-RP** | 0.28 (0.28–0.47) | 0.32 (0.32–0.50) | 0.30 (0.18–0.43) | 0.57 (0.48–0.67) |
| **PDRBD-RP** | 0.27 (0.27–0.47) | 0.31 (0.31–0.49) | 0.31 (0.19–0.44) | 0.55 (0.45–0.64) |
| **PD-RP** | 0.28 (0.28–0.48) | 0.32 (0.32–0.51) | 0.31 (0.19–0.44) | 0.57 (0.47–0.67) |
| **iRBDconv-RP** | 0.24 (0.24–0.42) | 0.28 (0.28–0.46) | 0.23 (0.12–0.34) | 0.56 (0.46–0.65) |

*Legend: conv = conversion; DLB = Dementia with Lewy Bodies; PD = Parkinson’s Disease; RBD = REM Sleep Behaviour Disorder. RP = Related Pattern.*


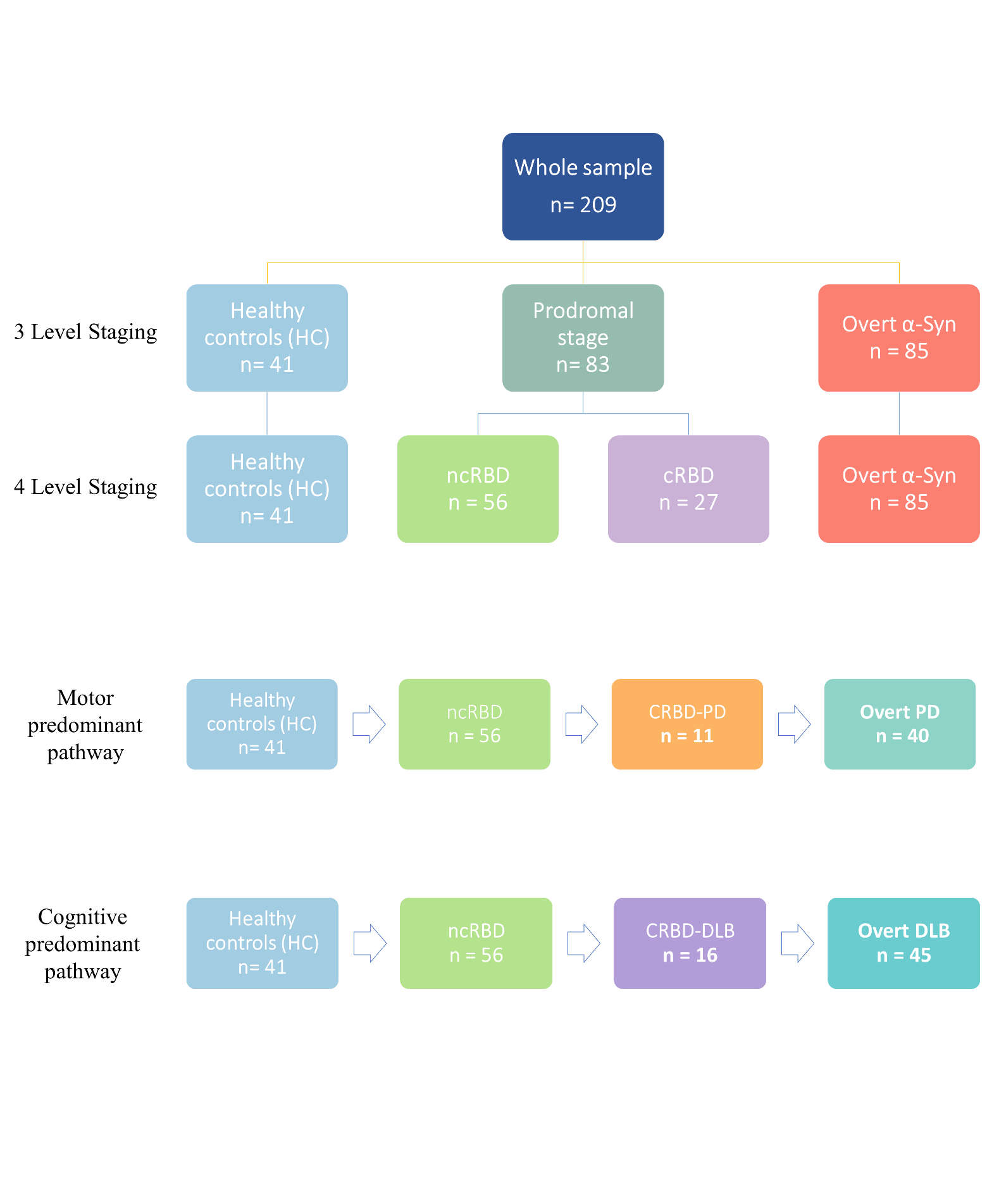
**Supplementary Figure 1:** Framework of granularity along the continuum from prodromal to overt alpha-synucleinopathy.

*Legend: cRBD = converters RBD; DLB = Dementia with Lewy Bodies; HC = Healthy Controls; ncRBD = non-converters RBD; PD = Parkinson’s Disease; RBD = REM Sleep Behaviour Disorder*


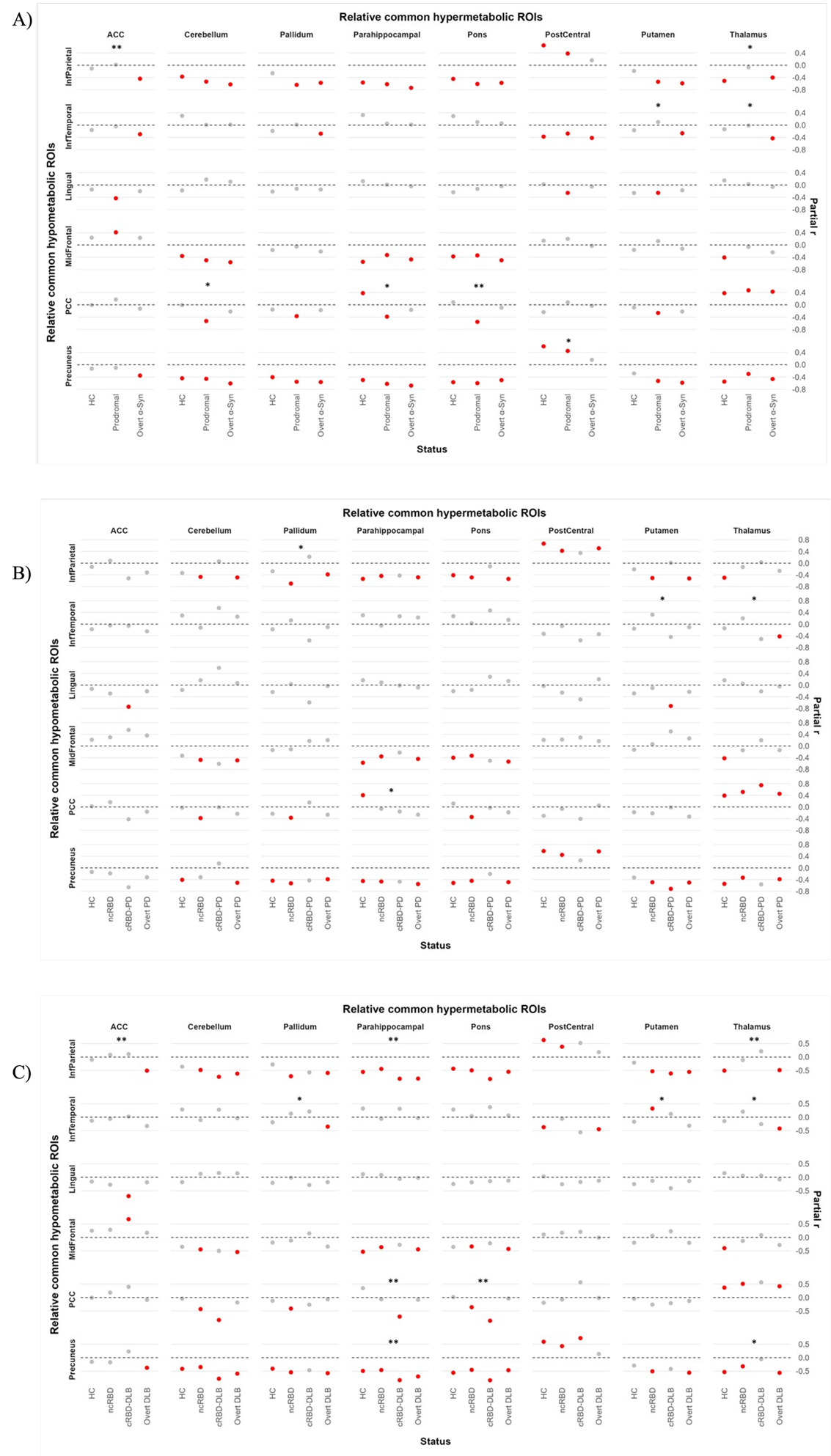
**Supplementary Figure 2:** Interregional partial correlations between common relative hyper- and hypo-metabolic SUVRs across disease groups of the A) 3-level stratification; B) Motor-predominant pathway; C) Cognitive-predominant pathway. Red dots represent significant correlations that survived FDR-correction for multiple comparisons (age- and sex-adjusted).

*Legend: *= uncorrected p<0.05 for interaction term; **= FDR-corrected p<0.05 for interaction term; α-Syn = α-synucleinopathy; cRBD = converters RBD; DLB = Dementia with Lewy Bodies; HC = Healthy Controls; ncRBD = non-converters RBD; PD = Parkinson’s Disease; RBD = REM Sleep Behaviour Disorder. ACC = Anterior Cingulate Cortex; Inf = Inferior; Mid =Middle; PCC = Posterior cingulate Cortex.*

# References

1. Mattioli P, Pardini M, Famà F, et al. Cuneus/precuneus as a central hub for brain functional connectivity of mild cognitive impairment in idiopathic REM sleep behavior patients. *Eur J Nucl Med Mol Imaging*. 2021;(Mci). doi:10.1007/s00259-021-05205-6

2. Litvan I, Goldman JG, Tröster AI, et al. Diagnostic criteria for mild cognitive impairment in Parkinson’s disease: Movement Disorder Society Task Force guidelines. *Mov Disord*. 2012;27(3):349-356.

3. Berry RB, Brooks R, Gamaldo C, et al. AASM scoring manual updates for 2017 (version 2.4). *J Clin Sleep Med*. 2017;13(5):665-666.

4. Yoon EJ, Lee J-Y, Kim H, et al. Brain metabolism related to mild cognitive impairment and phenoconversion in patients with isolated REM sleep behavior disorder. *Neurology*. 2022;98(24):e2413-e2424.

5. Lee J-Y, Yoon EJ, Kim YK, et al. Nonmotor and dopamine transporter change in REM sleep behavior disorder by olfactory impairment. *J Mov Disord*. 2019;12(2):103.

6. Frauscher B, Iranzo A, Gaig C, et al. Normative EMG values during REM sleep for the diagnosis of REM sleep behavior disorder. *Sleep*. 2012;35(6):835-847.

7. Orso B, Famà F, Giorgetti L, et al. Polysomnographic correlates of sleep disturbances in de novo, drug naïve Parkinson’s Disease. *Neurol Sci*. Published online 2021:1-6.

8. Spetsieris PG, Eidelberg D. Scaled subprofile modeling of resting state imaging data in Parkinson’s disease: methodological issues. *Neuroimage*. 2011;54(4):2899-2914.

9. Meles SK, Teune LK, de Jong BM, Dierckx RA, Leenders KL. Metabolic imaging in Parkinson disease. *J Nucl Med*. 2017;58(1):23-28.
